# Which Is the Best *In Silico* Program for the Missense Variations in IDUA Gene? A Comparison of 33 Programs Plus a Conservation Score and Evaluation of 586 Missense Variants

**DOI:** 10.3389/fmolb.2021.752797

**Published:** 2021-10-21

**Authors:** Pâmella Borges, Gabriela Pasqualim, Ursula Matte

**Affiliations:** ^1^ Cell, Tissue and Gene Laboratory, Clinicas Hospital of Porto Alegre (HCPA), Porto Alegre, Brazil; ^2^ Bioinformatics Core, Experimental Research Centre, HCPA, Porto Alegre, Brazil; ^3^ Graduate Programme in Genetics and Molecular Biology, Federal University of Rio Grande Do Sul (UFRGS), Porto Alegre, Brazil; ^4^ Genetics Laboratory, Biological Sciences Institute, Federal University of Rio Grande (FURG), Porto Alegre, Brazil; ^5^ Department of Genetics, UFRGS, Porto Alegre, Brazil

**Keywords:** mucopolysaccharidosis type I (MPS I), missense variants, in silico predictions, VUS classifications, molecular diagnosis

## Abstract

Mucopolysaccharidosis type I (MPS I) is an autosomal recessive disease characterized by the deficiency of alpha-L-iduronidase (*IDUA*), an enzyme involved in glycosaminoglycan degradation. More than 200 disease-causing variants have been reported and characterized in the *IDUA* gene. It also has several variants of unknown significance (VUS) and literature conflicting interpretations of pathogenicity. This study evaluated 586 variants obtained from the literature review, five population databases, in addition to dbSNP, Human Genome Mutation Database (HGMD), and ClinVar. For the variants described in the literature, two datasets were created based on the strength of the criteria. The stricter criteria subset had 108 variants with expression study, analysis of healthy controls, and/or complete gene sequence. The less stringent criteria subset had additional 52 variants found in the literature review, HGMD or ClinVar, and dbSNP with an allele frequency higher than 0.001. The other 426 variants were considered VUS. The two strength criteria datasets were used to evaluate 33 programs plus a conservation score. BayesDel (addAF and noAF), PON-P2 (genome and protein), and ClinPred algorithms showed the best sensitivity, specificity, accuracy, and kappa value for both criteria subsets. The VUS were evaluated with these five algorithms. Based on the results, 122 variants had total consensus among the five predictors, with 57 classified as predicted deleterious and 65 as predicted neutral. For variants not included in PON-P2, 88 variants were considered deleterious and 92 neutral by all other predictors. The remaining 124 did not obtain a consensus among predictors.

## Introduction

Mucopolysaccharidosis type I (MPS I) is an autosomal recessive disease characterized by the deficiency of alpha-L-iduronidase (*IDUA*) involved in glycosaminoglycan (GAG) degradation (Scott et al., 1991). This deficiency leads to progressive lysosomal accumulation of heparan and dermatan sulfate and causes a gradual deterioration of cells and tissues that culminate in early death in severe cases (Lehman et al., 2011). MPS I has a considerable phenotypic variation, with an extensive range of clinical manifestations and well-defined extreme phenotypes. Scheie syndrome (MPS I-S; OMIM# 607016) is the attenuated phenotype and includes somatic involvement, while Hurler syndrome (MPS I-H; OMIM# 607014) is the severe phenotype with important neurological impairment, among other features (Kubaski et al., 2020). All phenotypes exhibit excessive GAG accumulation and excretion in urine and are indistinguishable by routine biochemical tests (Lehman et al., 2011; Viana et al., 2011).

More than 200 disease-causing variants have been reported and characterized in the *IDUA* gene (Bertola et al., 2011). In a 2019 study with data from the MPS I Registry, non-sense and missense variants corresponded, respectively, to 56.5 and 33.6% of the reported variants (Clarke et al., 2019). Attenuated cases present at least one allele with residual activity, generally due to missense variants, regardless of the other alleles, and genotype–phenotype correlation has been established for some missense pathogenic variants (Fuller et al., 2005). Non-disease-causing missense variants, such as p.Arg105Gln, p.Gln63Pro (Scott et al., 1991), p.His33Gln (Scott et al., 1992), and p.Ala361Thr (Clarke and Scott, 1993), have also been described in the literature.

The broader use of massive parallel genetic sequencing increased the list of variants of unknown significance (VUS). Functional molecular assessments do not accompany the pace of detection of new genetic variants. Most variants present in the Exome Aggregation Consortium (ExAC) and Genome Aggregation Database (gnomAD) (Lek et al., 2016; Karczewski et al., 2020) have not yet been described or evaluated. Therefore, research and clinical laboratories use *in silico* strategies to help understand the biological significance of VUS. These methods are already considered in ACMG standard guidelines (Richards et al., 2015) to indicate some evidence level when clinical information is insufficient or non-existent. Clinical laboratories also created their guideline on variant interpretation, named Sherloc (semi-quantitative, hierarchical evidence-based rules for locus interpretation) (Nykamp et al., 2017).

Even though computational analysis is often used, results must be viewed with caution. Not only do different programs have discordant results for the same gene, but algorithms may also have different values of accuracy, specificity, and sensitivity depending on the characteristics of the gene or protein. Therefore, ideally, a performance assessment should be performed for each gene/protein to choose the best algorithm for variant prioritization. However, this also needs reliable standards as calibrators—literature and curated databases also show divergence.

*This study aims to compare in silico predictors using two datasets of variants with different degrees of confidence.* Using the best predictors indicated by these two datasets, we evaluated the VUS present in the *IDUA* gene in population databases.

## Materials and Methods

### Curated Variant Selection

We created a database with missense variants described in the literature, in curated databases, and in population databases with frequencies greater than 0.001. To perform such studies, a number of benign and pathogenic variants are needed, and they can only be obtained with comprehensive review of the literature; therefore, we opted for a single gene study. We performed a manual review of all missense variants in the *IDUA* gene published between 1991 and 2019. According to the variant classification methods in each manuscript, variants from the literature were divided into two subsets (strong or weak evidence). Evidence was considered strong if at least one of the following was performed: expression study, evaluation of healthy controls, or complete gene sequence corroborating the pathogenic or non-pathogenic disease-causing variant status. The subset of variants with weak criteria comprised all variants in the strong subset plus the rest of missense variants described in the literature, variants from the HGMD (Stenson et al., 2014) and ClinVar (with their classifications) (Landrum et al., 2014), and variants in population databases with allele frequencies greater than 0.001. These two subsets were selected to evaluate the prediction programs’ characteristics and to compare the correlation between variants’ predictions and literature information. Variants that do not have any of these criteria were considered VUS.

### *In Silico* Programs

We analyzed 33 prediction algorithms and one conservation score. For better comparison, all available training sets for each program were evaluated separately. We obtained prediction for SIFT (protein data training) (Kumar et al., 2009), SIFT4G (Vaser et al., 2016), PolyPhen2 (HDIV and HVAR) (Adzhubei et al., 2013), LRT (Chun and Fay, 2009), MutationTaster2 (Schwarz et al., 2010), MutationAssessor (Reva et al., 2007), FATHMM (Coding Variants-Weighted, MKL coding, and XF coding) (Shihab et al., 2013), MetaSVM/LR (Dong et al., 2015), CADD (GRCh37/hg19 and GRCh38/hg38) (Kircher et al., 2014), VEST4 (Carter et al., 2013), PROVEAN (protein data training) (Choi et al., 2012), fitCons x4 (Gulko et al., 2015), LINSIGHT (Huang et al., 2017), M-CAP (Jagadeesh et al., 2016), REVEL (Ioannidis et al., 2016), MutPred (Li et al., 2009), PrimateAI (Sundaram et al., 2019), BayesDel (addAF and noAF) (Feng, 2017), ClinPred (Alirezaie et al., 2018), and LIST-S2 (Malhis et al., 2020) prediction algorithms. We also tested the GERP++ conservation score (Davydov et al., 2010) from dbNSFP v4.1a, a database developed for functional prediction and annotation of all potential non-synonymous single-nucleotide variants (nsSNVs) in the human genome (Liu et al., 2020).

The predictions of PhD-SNP (Capriotti et al., 2006), PANTHER (Thomas et al., 2003), SNPs&GO (Capriotti et al., 2013), PredictSNP (Bendl et al., 2016), CADD 1.2 (Kircher et al., 2014), DANN (Quang et al., 2015), FATHMM (Coding Variants-Unweighted) (Shihab et al., 2013), FunSeq2 (Fu et al., 2014), GWAVAE 1.0 (Ritchie et al., 2014), SuSPect (Yates et al., 2014), PMUT (Ferrer-Costa et al., 2005), CONDEL (González-Pérez and López-Bigas, 2011), PROVEAN (genome data training) (Choi et al., 2012), SIFT (genome data training) (Kumar et al., 2009), PON-P2 (identifier, protein, and genome data training) (Niroula et al., 2015), and MutPred (Li et al., 2009) were obtained from the web-based application. The variant classifiers were used when provided by the algorithm. The scores of VEST4 (Carter et al., 2013), REVEL (Ioannidis et al., 2016), MutPred (Li et al., 2009), CADD_raw, CADD_phred (Kircher et al., 2014), integrated_fitCons (Gulko et al., 2015), SuSPect (Yates et al., 2014), and GERP++_NR (Davydov et al., 2010) were transformed in binary classification. The cutoff of 0.5 was applied for SuSPect (Yates et al., 2014) and VEST4 (Carter et al., 2013), 0.75 for MutPred (Li et al., 2009) and REVEL (Ioannidis et al., 2016), 20 for CADD_phred, zero for CADD_raw (Kircher et al., 2014), 0.4 for fitCons x4 (Gulko et al., 2015), and 0.047 for GERP++ (Davydov et al., 2010) as suggested by the authors.

### Variants of Unknown Significance

All missense variants in the canonical *IDUA* sequence present in ExAC v0.3.1 (Lek et al., 2016), gnomAD v2.0.2 (Karczewski et al., 2020), ABraOM (Naslavsky et al., 2017), LOVD (Fokkema et al., 2011), 1000 Genomes (1000 Genomes Project Consortium et al., 2015), and dbSNP (Sherry et al., 2001) with frequencies less than 0.0001, plus variants in the Human Genome Mutation Database (HGMD) (Stenson et al., 2014) and ClinVar (Landrum et al., 2014), were considered VUS. These variants were merged in a single database to remove duplicates and exclude those included in the datasets previously used to compare the algorithms.

### Statistical Analysis

The statistical analysis was performed using SPSS (Statistical Package for the Social Sciences) and python algorithms. The sensitivity, specificity, positive predictive value (PPV), negative predictive value (NPV), accuracy, true-positive rate (TPR), false-positive rate (FPR), and Fisher’s exact test were calculated on python with libraries matplotlib.pyplot (Hunter, 2007), sklearn.metrics (Pedregosa et al., 2011), pandas (Zenodo, 2020), and NumPy (Harris et al., 2020). The kappa value was generated with SPSS 18.03.

## Results

**FIGURE 1:**
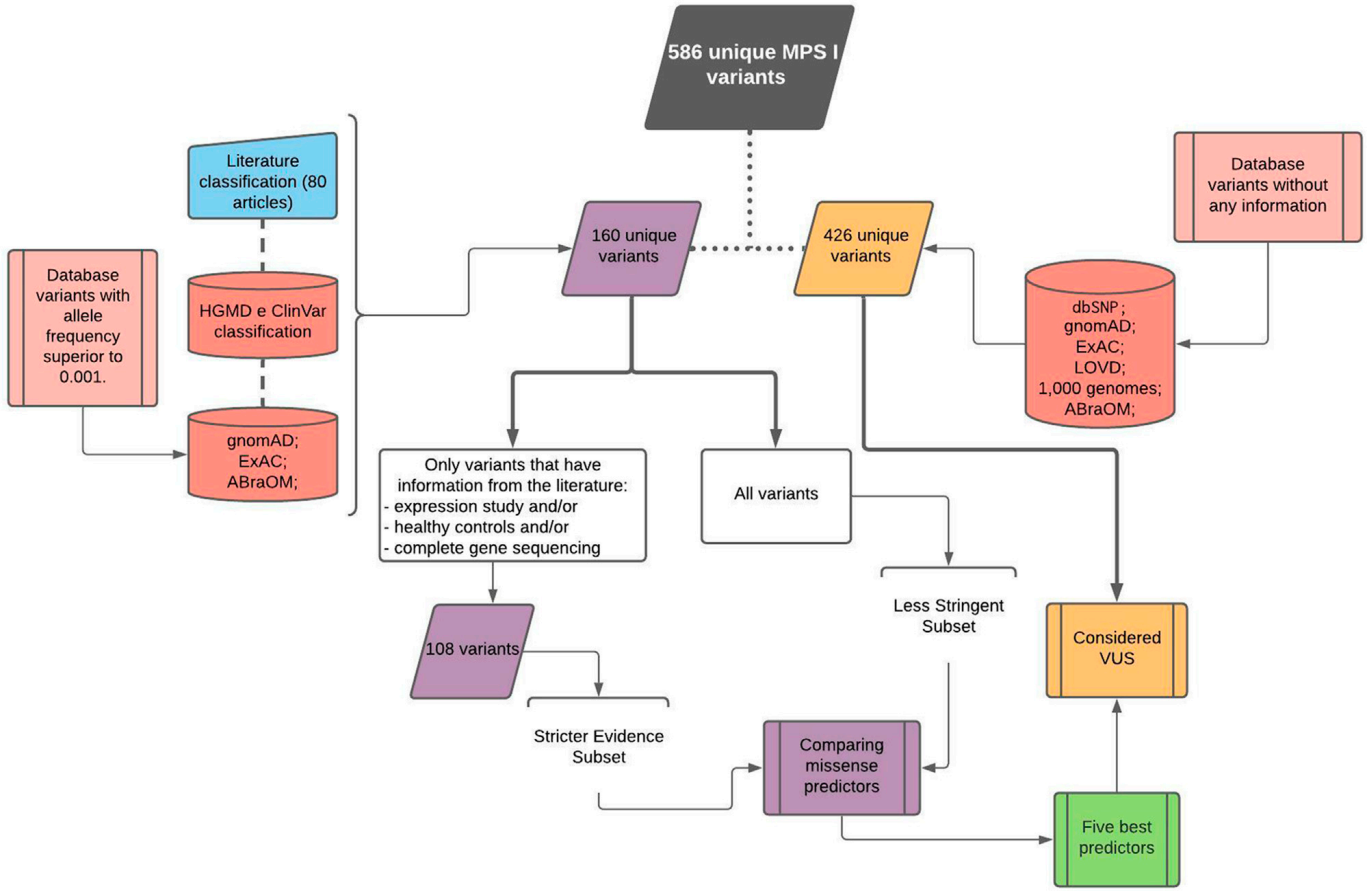
Workflow chart showing variant retrieval and curation.

A total of 586 unique variants were analyzed in this study obtained according to the workflow presented in Figure 1. Each database’s contribution can be seen in Supplementary Figure S1. dbSNP (Sherry et al., 2001) and gnomAD v2.0.2 (Karczewski et al., 2020) databases had the larger number of variants, 363 and 316, respectively, with 83 and 86 exclusive ones. ExAC v0.3.1 (Lek et al., 2016) contributed with 266 variants, with only six exclusive ones. LOVD (Fokkema et al., 2011) presented 44 variants, with three exclusive ones, whereas HGMD (Stenson et al., 2014) and ClinVar (Landrum et al., 2014) contributed with 3 and 19 exclusive variants, respectively, from a total of 136 and 131. ABraOM (Naslavsky et al., 2017) and 1000 Genomes (1000 Genomes Project Consortium et al., 2015) presented 19 and 47 variants, respectively, but none was private.

First, 145 variants manually retrieved from the literature were combined with variants in curated databases and population databases with frequencies higher than 0.001. This formed a set of 160 unique variants used to compare the algorithms. Another 426 variants were obtained from population databases and considered VUS.

**FIGURE 2:**
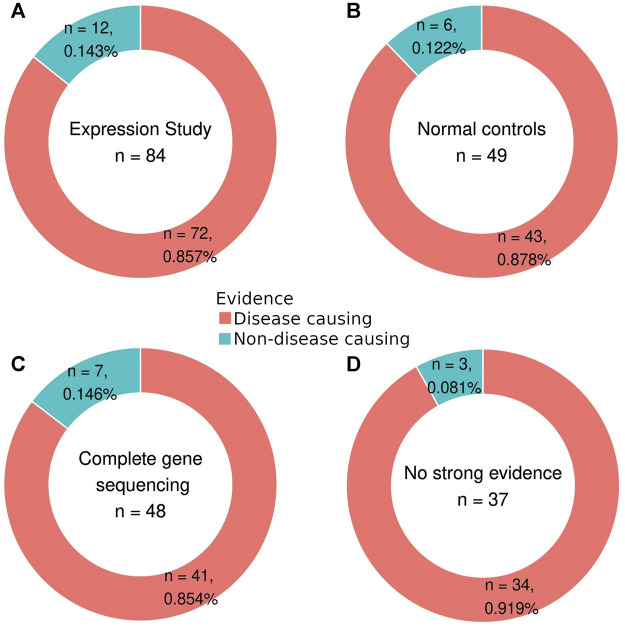
Percentage of disease-causing and non-disease-causing variants in each evidence criterion: variants with expression study **(A)**, comparison with normal controls **(B)**, complete gene sequencing **(C)**, and absence of strong evidence **(D)**.

According to the type of evidence used for their description, variants in the first set of 160 were divided into two subgroups. Out of the 145 variants from the literature, 108 had at least one of three measures that were considered strong evidence criteria (Figure 2). In this group of variants with strong evidence, 91 were disease-causing, and of these, 19 variants did not have expression studies, 48 variants were not analyzed in healthy controls, and 50 variants were not described in studies with complete gene sequencing (Supplementary Table S1). Of the 17 non-disease-causing variants in the group with strong evidence, only five were not analyzed by expression studies (Supplementary Table S2).

The 160 variants (26 benign and 134 pathogenic) in the weak criteria subset and 108 variants (17 benign and 91 pathogenic) in the strong criteria subset were used for evaluating 33 prediction algorithms plus one conservation score. As one program may present more than one training dataset, a total of 51 estimates were obtained. SIFT (Kumar et al., 2009), PROVEAN (Choi et al., 2012), PolyPhen2 (Adzhubei et al., 2013), BayesDel (Feng, 2017), CADD (Kircher et al., 2014), FATHMM (Shihab et al., 2013), fitCons (Gulko et al., 2015), MutPred (Li et al., 2009), and PON-P2 (Niroula et al., 2015) were evaluated for every available training set.

For the strong criteria subset, only BayesDel (addAF and noAF) (Feng, 2017), PON-P2 (genome, protein, and identifier) (Niroula et al., 2015), and ClinPred (Alirezaie et al., 2018) presented accuracy higher than 90% and kappa value higher than 0.6, with PON-P2 (genome database) (Niroula et al., 2015), ClinPred (Alirezaie et al., 2018), and BayesDel (addAF) (Feng, 2017) being the ones with the best relation between sensitivity and specificity and higher kappa values (0.692, 0.719, and 0.821) (Supplementary Table S3). One PPV could not be calculated because FunSeq2 (Fu et al., 2014) classified all variants as benign. Three algorithms (integrated_fitCons, GM12878_fitCons (Gulko et al., 2015), and M-CAP (Jagadeesh et al., 2016)) classified all variants as pathogenic and did not present an NPV. The kappa value also could not be calculated for these four predictors.

The smallest sensitivities (between 0 and 0.3) were observed in PrimateAI (Sundaram et al., 2019) and SuSPect (Yates et al., 2014) predictors. Excluding predictors that have maximum sensitivity and minimal specificity, the algorithms PolyPhen2 (HDIV) (Adzhubei et al., 2013), MutationTaster2 (Schwarz et al., 2010), MutationAssessor (Reva et al., 2007), VEST4 (Carter et al., 2013), BayesDel (addAF and noAF) (Feng, 2017), ClinPred (Alirezaie et al., 2018), CADD (raw_hg38, phred_hg38, raw_hg19, phred_hg19) (Kircher et al., 2014), FATHMM (Coding Variants-Weighted) (Shihab et al., 2013), H1hESC_fitCons (Gulko et al., 2015), GERP++ (Davydov et al., 2010), CONDEL (González-Pérez and López-Bigas, 2011), and PON-P2 (identifier, protein, and genome) (Niroula et al., 2015) presented large sensitivity (over 90%). Excluding FunSeq2 (Fu et al., 2014), only SNPs&GO (Capriotti et al., 2013) had specificity higher than 90%, and 14 algorithms had specificity between 80 and 90% (Supplementary Table S3).

**FIGURE 3:**
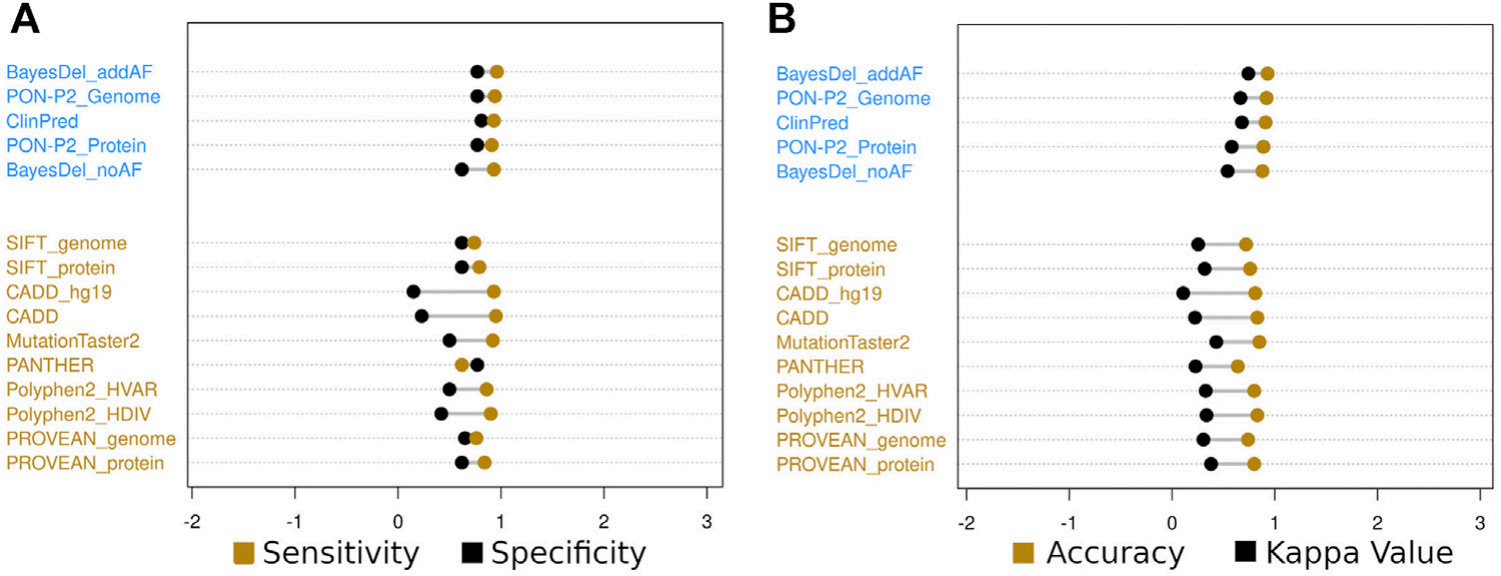
Sensitivity and specificity **(A)** and accuracy and kappa value **(B)** of the top five classifiers in blue (BayesDel-addAF, PON-P2-genome, ClinPred, PON-P2-protein, and BayesDel-noAF algorithms) and the top six cited in yellow (SIFT, CADD, MutationTaster2, PANTHER, PolyPhen2, and PROVEAN) for the less stringent criteria subset.

The weak criteria subset showed similar patterns to the strong criteria subset despite obtaining a general reduction in the calculated values, except for the PON-P2 (identifier) algorithm (Niroula et al., 2015) that showed an increased sensitivity. The same four algorithms classified all variants as only benign or pathogenic. In this subset, no algorithm had specificity higher than 90%, and nine algorithms had specificity between 80 and 90%, including PrimateAI (Sundaram et al., 2019) and SNPs&GO (Capriotti et al., 2013) (Supplementary Figures S2A,B). In this subset, PON-P2 (genome database) (Niroula et al., 2015), ClinPred (Alirezaie et al., 2018), and BayesDel (addAF) (Feng, 2017) obtained accuracy higher than 90% (0.92, 0.91, and 0.93) and kappa values higher than 0.6 (0.666, 0.680, and 0.743) (Figures 3A,B). All sensitivity, specificity, accuracy, PPV, NPV, FPR, and kappa values are displayed in Supplementary Tables S3,4 for the strong and weak criteria subsets.

**FIGURE 4:**
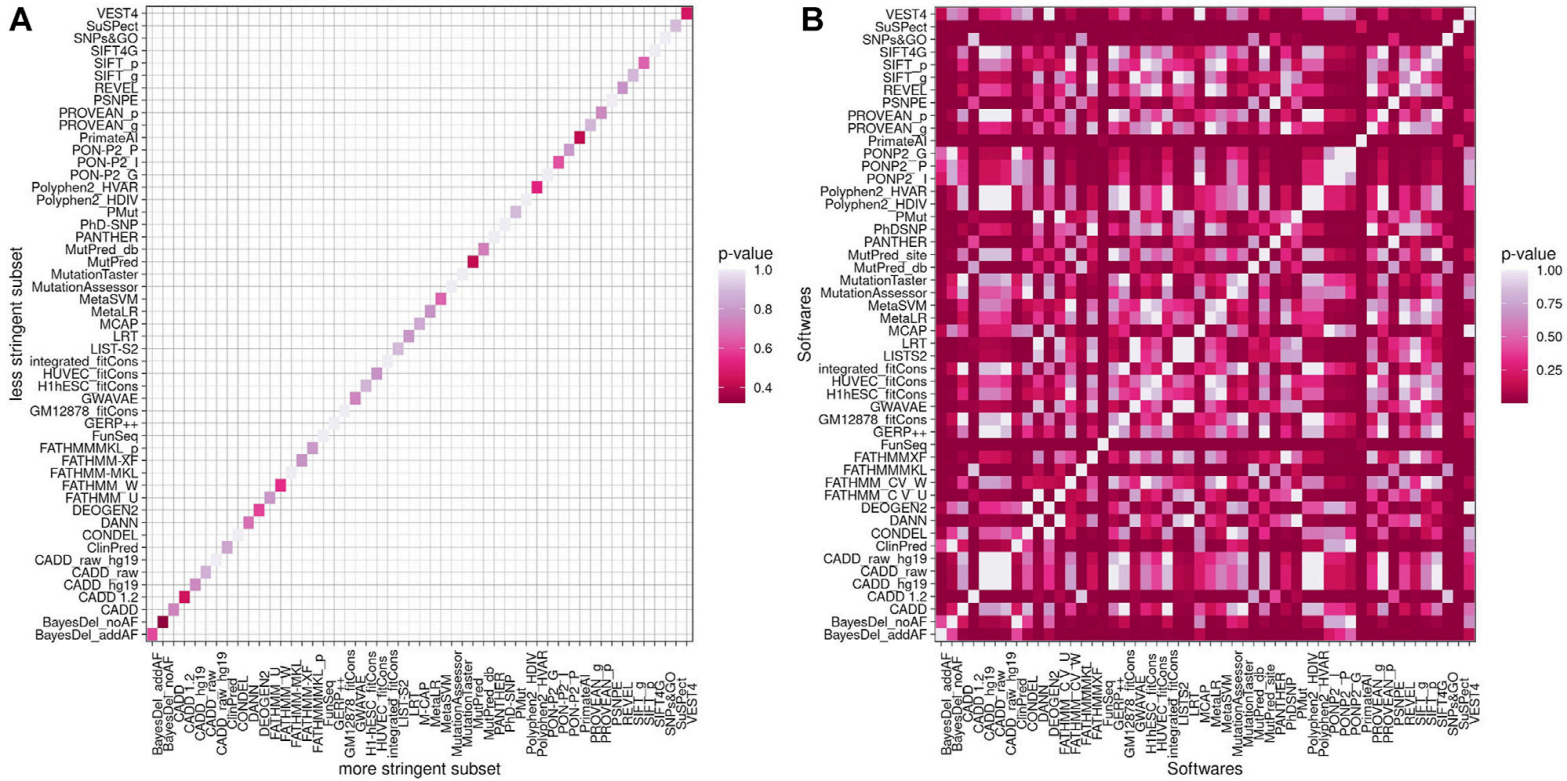
*p*-Value of Fisher’s exact test comparing less stringent criteria and more stringent criteria **(A)** and the 51 estimates in a less stringent subset **(B)**.

Fisher’s exact test was performed to test if weak and strong subsets present statistical differences in predictors’ performance. The ratio of hits and errors for each program was compared between weak and strong subsets, and none presented statistically significant values (Figure 4A). When we compared the same subset estimates, both subsets had the same pattern with several *p*-values lower than 0.05, as shown in Figure 4B for the weak criteria subset.

**FIGURE 5:**
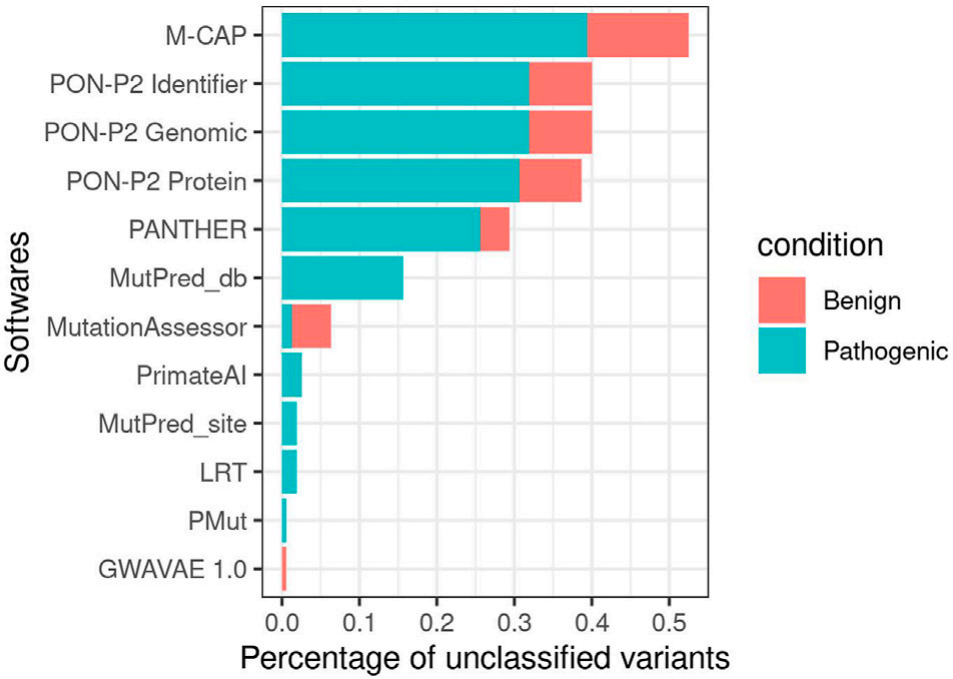
Number of unclassified variants per software for the less stringent criteria subset.

Not all 51 estimates were obtained for all 160 variants. MutationAssessor (Reva et al., 2007), LRT (Chun and Fay, 2009), PrimateAI (Sundaram et al., 2019), PANTHER (Thomas et al., 2003), GWAVAE (Ritchie et al., 2014), PMUT (Ferrer-Costa et al., 2005), M-CAP (Jagadeesh et al., 2016), MutPred (Li et al., 2009), and all three PON-P2 (Niroula et al., 2015) algorithms did not return a predicted classification for some variants (Figure 5). All three PON-P2 (Niroula et al., 2015) training sets were the predictors that contained the most unclassified variants, followed by MutPred (Li et al., 2009) and predictions obtained from dbNSFP (Liu et al., 2020). The algorithms LRT (Chun and Fay, 2009) (2), MutationAssessor (Reva et al., 2007) (3), and PrimateAI (Sundaram et al., 2019) (3) failed to classify variants in the first amino acid (MutationAssessor (Reva et al., 2007) and PrimateAI (Sundaram et al., 2019)) or at the end of the protein (LRT (Chun and Fay, 2009)).

For the strong criteria subset, all programs failed to report more pathogenic variants except for M-CAP (Jagadeesh et al., 2016). MutationAssessor (Reva et al., 2007), PrimateAI (Sundaram et al., 2019), and PANTHER (Thomas et al., 2003) presented the fewest number of unclassified variants, which are only pathogenic. MutPred (Li et al., 2009) dbNSFP (Liu et al., 2020) produced a larger number of unclassified variants that are both benign and pathogenic. For the weak criteria subset, MutPred (Li et al., 2009) and dbNSFP (Liu et al., 2020) increased the number of unclassified variants, exceeding the other programs (Figure 4). LRT (Chun and Fay, 2009) and PMUT (Ferrer-Costa et al., 2005) had one benign and one pathogenic uncategorized variant, respectively, in this subset. M-CAP (Jagadeesh et al., 2016) continued to show more benign (8) than pathogenic (2) variants unclassified.

### *In Silico* VUS Classification

**FIGURE 6:**
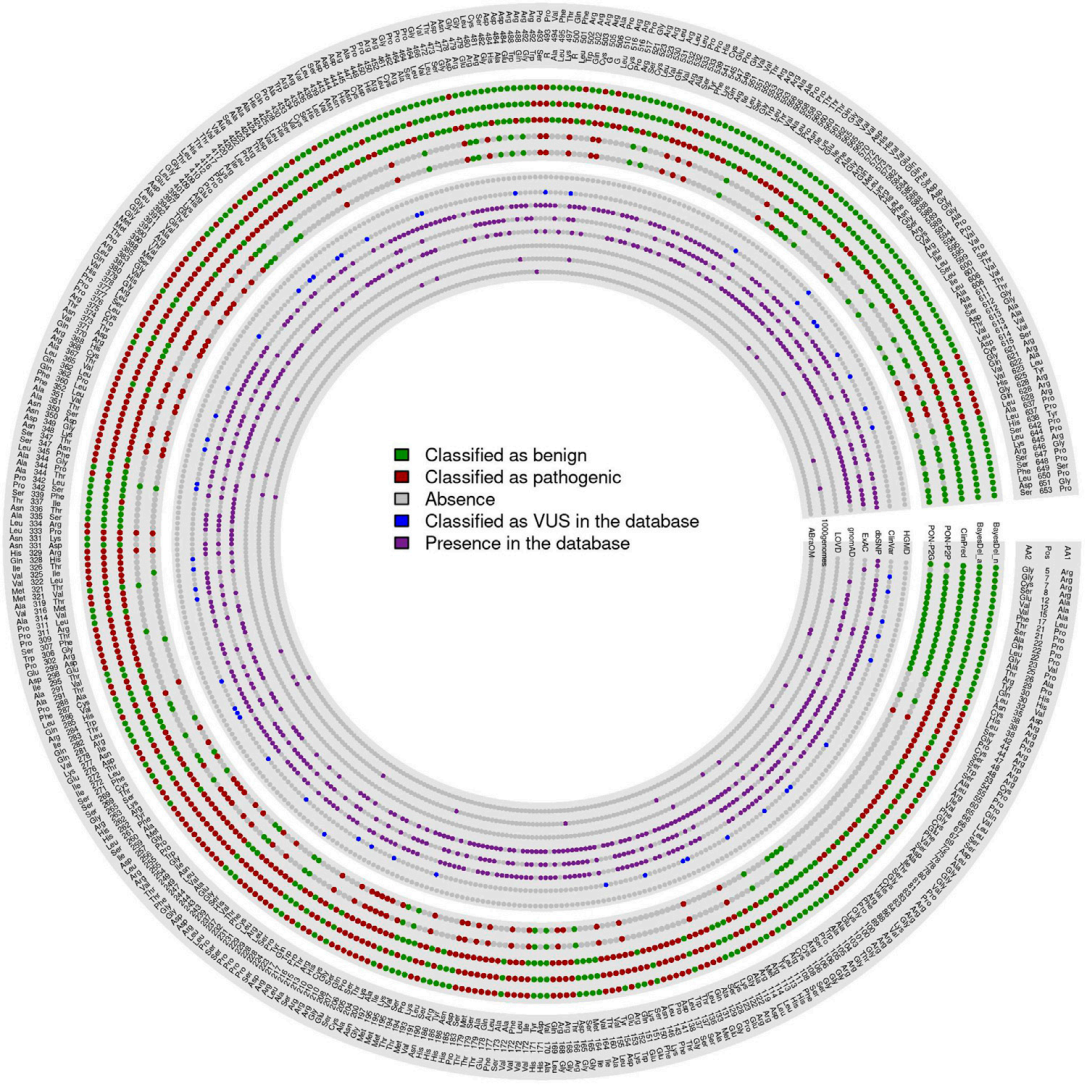
VUS classified by all best software.

Based on values present in both evaluation subsets, the 426 VUS were classified using the best five predictors: BayesDel (addAF and noAF) (Feng, 2017), PON-P2 (genome and protein) (Niroula et al., 2015), and ClinPred algorithms (Alirezaie et al., 2018). PON-P2 (genome and protein) (Niroula et al., 2015) is the only of these five predictors that do not classify every variant, with both failing to classify 267 variants plus six unclassified variants exclusive to PON-P2-genome (Niroula et al., 2015) and other six exclusive to PON-P2-protein (Niroula et al., 2015). Out of the 426 variants, 57 obtained a total consensus of the five programs as pathogenic and 65 as benign. For variants not included in PON-P2 (Niroula et al., 2015), 88 variants were considered pathogenic and 92 benign by all other predictors. The remaining 124 did not obtain a consensus among predictors (Figure 6).

## Discussion

In this study, we evaluated the prediction of 33 programs plus a conservation score for missense variants in the *IDUA* gene. Two datasets were created based on literature information and public databases: The first dataset was used to evaluate the best response predictors for missense *IDUA* variants. The second dataset comprised 426 VUS that were evaluated by the five best-performing algorithms. For the first dataset, two subsets were separated based on standards: modifications with specific literature information as a strong criteria dataset and all variants present in literature review plus databases with variant classification and high allele frequency. These variants were included to increase the amount of non-disease-causing mutations in the curated dataset.

The subsets did not demonstrate a notable difference, although the weak criteria subset presented lower overall values. The difference in performance may be explained by the lower classification confidence of the weak criteria subset. While the strong criteria subsets represent a supervised subset and include variants with a high confidence level of categorization, the weak and more flexible subset may contain incorrect classification. That may be due to the relatively small number of variants introduced in the weak subset (52 added to 108 in the strong subset).

Despite that, both comparison groups present the same predictors with the most satisfactory performances. BayesDel (Feng, 2017), the best performance predictor, is a met-score that combines deleteriousness predictors in the naïve Bayesian approach and uses ClinVar (Landrum et al., 2014) variants as a standard to determine the cutoff value. For this predictor, the set that integrates maximum and minor allele frequencies across populations (addAF) presents superior performance to that without allele frequencies (noAF) (Feng, 2017). ClinPred (Alirezaie et al., 2018) had the second-highest value in the kappa test and used ClinVar (Landrum et al., 2014) as a training dataset and combined two machine learning algorithms: random forest (cforest) and gradient boosted decision tree (xgboost) models (Alirezaie et al., 2018). PON-P2 uses variation data from VariBench to train a random forest selection features predictor for pathogenicity association of amino acid substitutions and accept variations in multiple formats (Niroula et al., 2015). The primer format (protein) is the most responsive, despite presenting a more modest performance than the genome format.

Classic and often used predictors such as SIFT (genome and protein) (Kumar et al., 2009) and PolyPhen2 (HumDiv and HumVar) (Adzhubei et al., 2013) did not perform well in both comparison subsets. For the strong criteria subset, PolyPhen2 (HDIV) (Adzhubei et al., 2013), preferred for evaluating rare alleles, had good sensitivity (90%), accuracy (83%), and kappa value (0.372) but specificity lower than 50% (Supplementary Table S3). The CADD (Combined Annotation Dependent Depletion) score integrates multiple annotations into one metric (Kircher et al., 2014) and presents sensitivity higher than 90% and accuracy higher than 80% for GRCh37/hg19 and GRCh38/hg38. Unfortunately, it possessed one of the smallest specificities and kappa value between evaluated programs. A recently developed program, REVEL, an ensemble method that manages random forest (Ioannidis et al., 2016), displays a compelling performance, despite not being one of the best ones, with higher specificity (88%) than sensitivity (75%).

Several predictors use ClinVar (Landrum et al., 2014) and HGMD (Stenson et al., 2014) databases as training datasets. Therefore, some hits in our datasets are reanalysis of training variants and not an accurate interpretation of pathogenicity, but this is not the case for all evaluated variants. Also, it is not likely that this would bias our analysis, even though we worked with variants native to these databases (Figure 2), as the training datasets used for these programs incorporate many more variants in numerous genes.

A recurrent problem in performance evaluation is the disproportionality of training and evaluation sets regarding the number of benign and pathogenic variants, a discrepancy also found in our datasets. We observed a minimal absolute difference between the properties of pathogenic and benign modifications, with the strong criteria subset having 15.74% of benign variants while the weak criteria subset had 16.25%. This minor difference demonstrates the difficulty of obtaining benign variants for composing sets, even implementing more comprehensive standards to evaluate these *in silico* predictors. It also reflects the fact that *in silico* software is mostly trained with disease-causing variants, which may cause a bias in the analysis. That was shown by Niroula and Vihinen (2019), who compared ten predictors with a large set of non-pathogenic variants only and found specificity over 80% in just three predictors (PON-P2 (Niroula et al., 2015), VEST (Carter et al., 2013), and FATHMM (Shihab et al., 2013)). In our study, despite both subsets presenting various programs with high specificity, the proportion of pathogenic and benign variants does not allow for a proper evaluation of specificity or to state which programs would exhibit significant differences in performance in a set of more benign variants.

This study does not replace the ACMG (Richards et al., 2015) or Sherloc (Nykamp et al., 2017) standards and guidelines. However, it increases confidence in one stage of the classification process (computational predictive programs), mainly when used in the absence of additional clinical information, as is the case of variants deposited in public databases. As we do not have access to any clinical information about the 426 variants identified in the public databases, these guidelines could not be applied. Therefore, we used only the classification given by the best five predictors previously selected. A classification of 122 variants (57 pathogenic and 65 benign variants) was obtained with a total consensus of the five programs. The other 304 variants were unclassified by PON-P2 (Niroula et al., 2015) or did not reach an agreement. If PON-P2 (Niroula et al., 2015) was excluded, then 311 variants reached a consensus (pathogenic and benign).

The difference between the number of variants with and without consensus is common and represents a recurrent finding when only information from computational predictive programs is available. This disagreement is probably caused by the metrics used by each predictor and can be a problem when no literature-based validation exists for that particular gene and predictor.

## Conclusion

Variants in the *IDUA* gene were evaluated by 33 prediction algorithms and one conservation score for all available training sets. Two subsets were created using strong and weak criteria based on literature information available for each variant. The subsets demonstrated a small difference, with reduced values in the weak criteria subset but the same most accurate predictors. The five most significant predictors were used for evaluating 426 VUS obtained from public databases. Of these, 122 variants showed a total consensus of programs with high confidence in classification. The classification of the other 304 variants depends if researchers accept or not a reduction of confidence in classification using a simple consensus.

## Data Availability

The original contributions presented in the study are included in the article/Supplementary Material, and further inquiries can be directed to the corresponding author.
