# Supplementary figures and images for: Which Is the Best In Silico Program for the Missense Variations in IDUA Gene? A Comparison of 33 Programs Plus a Conservation Score and Evaluation of 586 Missense Variants
Source: Front Mol Biosci. 2021 Oct 21;8:752797. doi: 10.3389/fmolb.2021.752797 (PMC8566697; doi:10.3389/fmolb.2021.752797)

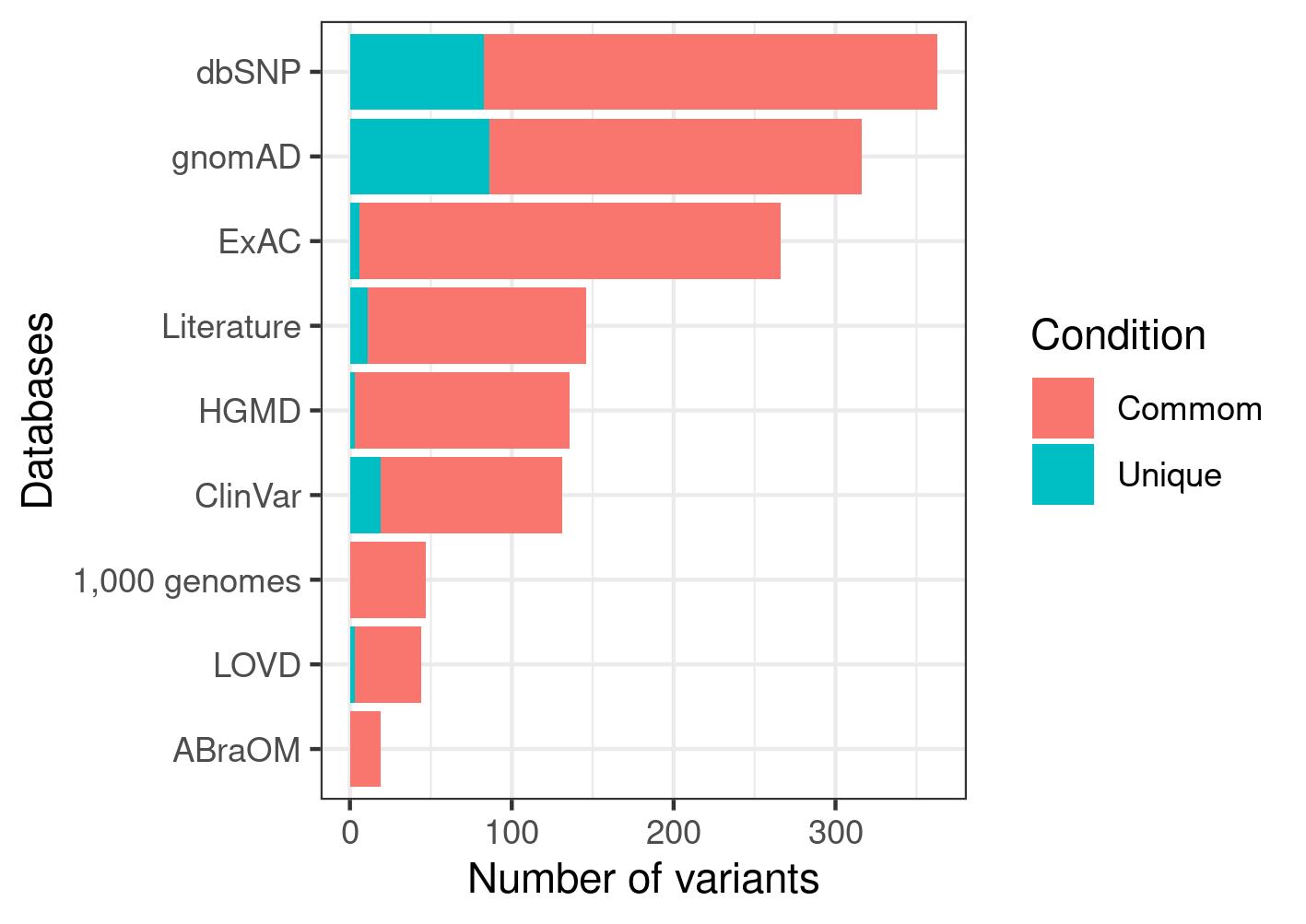

Supplement: Supplementary file 1 [file Image1.JPEG]
