# Supplementary material for: Which Is the Best In Silico Program for the Missense Variations in IDUA Gene? A Comparison of 33 Programs Plus a Conservation Score and Evaluation of 586 Missense Variants
Source: Front Mol Biosci. 2021 Oct 21;8:752797. doi: 10.3389/fmolb.2021.752797 (PMC8566697; doi:10.3389/fmolb.2021.752797)
Supplement: Supplementary file 2 [file DataSheet2.docx]

**References supplementary table 1 and 2**

Amr, K., Katoury, A., Abdel-Hamid, M., Bassiouni, R., Ibrahim, M., & Fateen, E. (2009). Mutational Analysis of the alpha-L-iduronidase gene in three Egyptian families: identification of three novel mutations and five novel polymorphisms. Genetic testing and molecular biomarkers, 13(6), 761–764. <https://doi.org/10.1089/gtmb.2009.0057>

Aronovich, E. L., Pan, D., & Whitley, C. B. (1996). Molecular genetic defect underlying alpha-L-iduronidase pseudodeficiency. American journal of human genetics, 58(1), 75–85.

Atçeken, N., Özgül, R. K., Yücel Yilmaz, D., Tokatli, A., Coşkun, T., Sivri, H. S., Dursun, A., & Karaca, M. (2016). Evaluation and identification of IDUA gene mutations in Turkishpatients with mucopolysaccharidosis type I. Turkish journal of medical sciences, 46(2), 404–408. <https://doi.org/10.3906/sag-1411-160>

Bach, G., Moskowitz, S. M., Tieu, P. T., Matynia, A., & Neufeld, E. F. (1993). Molecular analysis of Hurler syndrome in Druze and Muslim Arab patients in Israel: multiple allelic mutations of the IDUA gene in a small geographic area. American journal of human genetics, 53(2), 330–338.

Beesley, C. E., Meaney, C. A., Greenland, G., Adams, V., Vellodi, A., Young, E. P., & Winchester, B. G. (2001). Mutational analysis of 85 mucopolysaccharidosis type I families: frequency of known mutations, identification of 17 novel mutations and in vitro expression of missense mutations. Human genetics, 109(5), 503–511. <https://doi.org/10.1007/s004390100606>

Bravo, H., Neto, E. C., Schulte, J., Pereira, J., Filho, C. S., Bittencourt, F., Sebastião, F., Bender, F., de Magalhães, A., Guidobono, R., Trapp, F. B., Michelin-Tirelli, K., Souza, C., Rojas Málaga, D., Pasqualim, G., Brusius-Facchin, A. C., & Giugliani, R. (2017). Investigation of newborns with abnormal results in a newborn screening program for four lysosomal storage diseases in Brazil. Molecular genetics and metabolism reports, 12, 92–97. <https://doi.org/10.1016/j.ymgmr.2017.06.006>

Brooks, D. A., Fabrega, S., Hein, L. K., Parkinson, E. J., Durand, P., Yogalingam, G., Matte, U., Giugliani, R., Dasvarma, A., Eslahpazire, J., Henrissat, B., Mornon, J. P., Hopwood, J. J., & Lehn, P. (2001). Glycosidase active site mutations in human alpha-L-iduronidase. Glycobiology, 11(9), 741–750. <https://doi.org/10.1093/glycob/11.9.741>

Bunge, S., Clements, P. R., Byers, S., Kleijer, W. J., Brooks, D. A., & Hopwood, J. J. (1998). Genotype-phenotype correlations in mucopolysaccharidosis type I using enzyme kinetics, immunoquantification and in vitro turnover studies. Biochimica et biophysica acta, 1407(3), 249–256. <https://doi.org/10.1016/s0925-4439(98)00046-5>

Bunge, S., Kleijer, W. J., Steglich, C., Beck, M., Schwinger, E., & Gal, A. (1995). Mucopolysaccharidosis type I: identification of 13 novel mutations of the alpha-L-iduronidase gene. Human mutation, 6(1), 91–94. <https://doi.org/10.1002/humu.1380060119>

Bunge, S., Kleijer, W. J., Steglich, C., Beck, M., Zuther, C., Morris, C. P., Schwinger, E., Hopwood, J. J., Scott, H. S., & Gal, A. (1994). Mucopolysaccharidosis type I: identification of 8 novel mutations and determination of the frequency of the two common alpha-L-iduronidase mutations (W402X and Q70X) among European patients. Human molecular genetics, 3(6), 861–866. <https://doi.org/10.1093/hmg/3.6.861>

Burton, B. K., Charrow, J., Hoganson, G. E., Waggoner, D., Tinkle, B., Braddock, S. R., Schneider, M., Grange, D. K., Nash, C., Shryock, H., Barnett, R., Shao, R., Basheeruddin, K., & Dizikes, G. (2017). Newborn Screening for Lysosomal Storage Disorders in Illinois: The Initial 15-Month Experience. The Journal of pediatrics, 190, 130–135. <https://doi.org/10.1016/j.jpeds.2017.06.048>

Chistiakov, D. A., Savost'anov, K. V., Kuzenkova, L. M., Gevorkyan, A. K., Pushkov, A. A., Nikitin, A. G., Pakhomov, A. V., Vashakmadze, N. D., Zhurkova, N. V., Podkletnova, T. V., Mayansky, N. A., Namazova-Baranova, L. S., & Baranov, A. A. (2014). Molecular characteristics of patients with glycosaminoglycan storage disorders in Russia. Clinica chimica acta; international journal of clinical chemistry, 436, 112–120. <https://doi.org/10.1016/j.cca.2014.05.010>

Chkioua, L., Khedhiri, S., Kassab, A., Bibi, A., Ferchichi, S., Froissart, R., Vianey-Saban, C., Laradi, S., & Miled, A. (2011). Molecular analysis of mucopolysaccharidosis type I in Tunisia: identification of novel mutation and eight Novel polymorphisms. Diagnostic pathology, 6, 39. <https://doi.org/10.1186/1746-1596-6-39>

Chuang, C. K., Lin, H. Y., Wang, T. J., Huang, Y. H., Chan, M. J., Liao, H. C., Lo, Y. T., Wang, L. Y., Tu, R. Y., Fang, Y. Y., Chen, T. L., Ho, H. C., Chiang, C. C., & Lin, S. P. (2018). Status of newborn screening and follow up investigations for Mucopolysaccharidoses I and II in Taiwan. Orphanet journal of rare diseases, 13(1), 84. <https://doi.org/10.1186/s13023-018-0816-4>

Clarke, L. A., Nelson, P. V., Warrington, C. L., Morris, C. P., Hopwood, J. J., & Scott, H. S. (1994). Mutation analysis of 19 North American mucopolysaccharidosis type I patients: identification of two additional frequent mutations. Human mutation, 3(3), 275–282. <https://doi.org/10.1002/humu.1380030316>

Clarke, L. A., & Scott, H. S. (1993). Two novel mutations causing mucopolysaccharidosis type I detected by single strand conformational analysis of the alpha-L-iduronidase gene. Human molecular genetics, 2(8), 1311–1312. <https://doi.org/10.1093/hmg/2.8.1311>

Cobos, P. N., Steglich, C., Santer, R., Lukacs, Z., & Gal, A. (2015). Dried blood spots allow targeted screening to diagnose mucopolysaccharidosis and mucolipidosis. JIMD reports, 15, 123–132. <https://doi.org/10.1007/8904_2014_308>

Delgado Luengo, W. N., Miranda Contreras, L. E., Chávez, C. J., Solis-Añez, E., & Cammarata-Scalisi, F. (2014). Mutation c.1190-1delG/N in intron 8 and c.1708G>C/N in exon 12 not reported in the IDUA gene developed a clinical phenotype of Scheie syndrome. Investigacion clinica, 55(4), 365–370.

Dou, W., Peng, C., Zheng, J. K., & Gu, X. F. (2007). Zhonghua yi xue yi chuan xue za zhi = Zhonghua yixue yichuanxue zazhi = Chinese journal of medical genetics, 24(2), 136–139.

Furukawa, Y., Hamaguchi, A., Nozaki, I., Iizuka, T., Sasagawa, T., Shima, Y., Demura, S., Murakami, H., Kawahara, N., Okuyama, T., Iwasa, K., & Yamada, M. (2011). Cervical pachymeningeal hypertrophy as the initial and cardinal manifestation of mucopolysaccharidosis type I in monozygotic twins with a novel mutation in the alpha-L-iduronidase gene. Journal of the neurological sciences, 302(1-2), 121–125. <https://doi.org/10.1016/j.jns.2010.11.022>

Ghosh, A., Mercer, J., Mackinnon, S., Yue, W. W., Church, H., Beesley, C. E., Broomfield, A., Jones, S. A., & Tylee, K. (2017). IDUA mutational profile and genotype-phenotype relationships in UK patients with Mucopolysaccharidosis Type I. Human mutation, 38(11), 1555–1568. <https://doi.org/10.1002/humu.23301>

Guffon, N., Souillet, G., Maire, I., Straczek, J., & Guibaud, P. (1998). Follow-up of nine patients with Hurler syndrome after bone marrow transplantation. The Journal of pediatrics, 133(1), 119–125. <https://doi.org/10.1016/s0022-3476(98)70201-x>

Kamranjam, M., & Alaei, M. (2019). Mutation Analysis of the IDUA Gene in Iranian Patients with Mucopolysaccharidosis Type 1: Identification of Four Novel Mutations. Genetic testing and molecular biomarkers, 23(8), 515–522. <https://doi.org/10.1089/gtmb.2019.0022>

Kwak, M. J., Huh, R., Kim, J., Park, H. D., Cho, S. Y., & Jin, D. K. (2016). Report of 5 novel mutations of the α-L-iduronidase gene and comparison of Korean mutations in relation with those of Japan or China in patients with mucopolysaccharidosis I. BMC medical genetics, 17(1), 58. <https://doi.org/10.1186/s12881-016-0319-x>

Langereis, E. J., van den Berg, I., Halley, D., Poorthuis, B., Vaz, F. M., Wokke, J., & Linthorst, G. E. (2013). Considering Fabry, but Diagnosing MPS I: Difficulties in the Diagnostic Process. JIMD reports, 9, 117–120. <https://doi.org/10.1007/8904_2012_189>

Laradi, S., Tukel, T., Erazo, M., Shabbeer, J., Chkioua, L., Khedhiri, S., Ferchichi, S., Chaabouni, M., Miled, A., & Desnick, R. J. (2005). Mucopolysaccharidosis I: Alpha-L-Iduronidase mutations in three Tunisian families. Journal of inherited metabolic disease, 28(6), 1019–1026. <https://doi.org/10.1007/s10545-005-0197-4>

Lee, I. J., Hwang, S. H., Jeon, B. H., Song, S. M., Kim, J. S., Paik, K. H., Kwon, E. K., & Jin, D. K. (2004). Mutational analysis of the alpha-L-iduronidase gene in 10 unrelated Korean type I mucopolysaccharidosis patients: Identification of four novel mutations. Clinical genetics, 66(6), 575–576. <https://doi.org/10.1111/j.1399-0004.2004.00374.x>

Lee-Chen, G. J., Lin, S. P., Chen, I. S., Chang, J. H., Yang, C. W., & Chin, Y. W. (2002). Mucopolysaccharidosis type I: Identification and characterization of mutations affecting alpha-L-iduronidase activity. Journal of the Formosan Medical Association = Taiwan yi zhi, 101(6), 425–428.

Lee-Chen, G. J., Lin, S. P., Tang, Y. F., & Chin, Y. W. (1999). Mucopolysaccharidosis type I: characterization of novel mutations affecting alpha-L-iduronidase activity. Clinical genetics, 56(1), 66–70. <https://doi.org/10.1034/j.1399-0004.1999.560109.x>

Lee-Chen, G. J., & Wang, T. R. (1997). Mucopolysaccharidosis type I: identification of novel mutations that cause Hurler/Scheie syndrome in Chinese families. Journal of medical genetics, 34(11), 939–941. <https://doi.org/10.1136/jmg.34.11.939>

Li, P., Wood, T., & Thompson, J. N. (2002). Diversity of mutations and distribution of single nucleotide polymorphic alleles in the human alpha-L-iduronidase (IDUA) gene. Genetics in medicine : official journal of the American College of Medical Genetics, 4(6), 420–426. <https://doi.org/10.1097/00125817-200211000-00004>

Lin, S. P., Lin, H. Y., Wang, T. J., Chang, C. Y., Lin, C. H., Huang, S. F., Tsai, C. C., Liu, H. L., Keutzer, J., & Chuang, C. K. (2013). A pilot newborn screening program for Mucopolysaccharidosis type I in Taiwan. Orphanet journal of rare diseases, 8, 147. <https://doi.org/10.1186/1750-1172-8-147>

Matte, U., Yogalingam, G., Brooks, D., Leistner, S., Schwartz, I., Lima, L., Norato, D. Y., Brum, J. M., Beesley, C., Winchester, B., Giugliani, R., & Hopwood, J. J. (2003). Identification and characterization of 13 new mutations in mucopolysaccharidosis type I patients. Molecular genetics and metabolism, 78(1), 37–43. <https://doi.org/10.1016/s1096-7192(02)00200-7>

Oussoren, E., Keulemans, J., van Diggelen, O. P., Oemardien, L. F., Timmermans, R. G., van der Ploeg, A. T., & Ruijter, G. J. (2013). Residual α-L-iduronidase activity in fibroblasts of mild to severe Mucopolysaccharidosis type I patients. Molecular genetics and metabolism, 109(4), 377–381. <https://doi.org/10.1016/j.ymgme.2013.05.016>

Pasqualim, G., Ribeiro, M. G., da Fonseca, G. G., Szlago, M., Schenone, A., Lemes, A., Rojas, M. V., Matte, U., & Giugliani, R. (2015). p.L18P: a novel IDUA mutation that causes a distinct attenuated phenotype in mucopolysaccharidosis type I patients. Clinical genetics, 88(4), 376–380. <https://doi.org/10.1111/cge.12507>

Pineda, T., Marie, S., Gonzalez, J., García, A. L., Acosta, A., Morales, M., Correa, L. N., Vivas, R., Escobar, X., Protzel, A., Barba, M., Ospina, S., Corredor, C., Mansilla, S., & Velasco, H. M. (2014). Genotypic and bioinformatic evaluation of the alpha-l-iduronidase gene and protein in patients with mucopolysaccharidosis type I from Colombia, Ecuador and Peru. Molecular genetics and metabolism reports, 1, 468–473. <https://doi.org/10.1016/j.ymgmr.2014.10.001>

Pollard, L. M., Jones, J. R., & Wood, T. C. (2013). Molecular characterization of 355 mucopolysaccharidosis patients reveals 104 novel mutations. Journal of inherited metabolic disease, 36(2), 179–187. <https://doi.org/10.1007/s10545-012-9533-7>

Prommajan, K., Ausavarat, S., Srichomthong, C., Puangsricharern, V., Suphapeetiporn, K., & Shotelersuk, V. (2011). A novel p.E276K IDUA mutation decreasing α-L-iduronidase activity causes mucopolysaccharidosis type I. Molecular vision, 17, 456–460.

Raiman, J., & D'Aco, K. (2014). An 8-year-old girl with a history of stiff and painful joints. Pediatric annals, 43(8), 307–309. <https://doi.org/10.3928/00904481-20140723-05>

Scott, C. R., Elliott, S., Buroker, N., Thomas, L. I., Keutzer, J., Glass, M., Gelb, M. H., & Turecek, F. (2013). Identification of infants at risk for developing Fabry, Pompe, or mucopolysaccharidosis-I from newborn blood spots by tandem mass spectrometry. The Journal of pediatrics, 163(2), 498–503. <https://doi.org/10.1016/j.jpeds.2013.01.031>

Scott, H. S., Bunge, S., Gal, A., Clarke, L. A., Morris, C. P., & Hopwood, J. J. (1995). Molecular genetics of mucopolysaccharidosis type I: diagnostic, clinical, and biological implications. Human mutation, 6(4), 288–302. <https://doi.org/10.1002/humu.1380060403>

Scott, H. S., Litjens, T., Nelson, P. V., Thompson, P. R., Brooks, D. A., Hopwood, J. J., & Morris, C. P. (1993). Identification of mutations in the alpha-L-iduronidase gene (IDUA) that cause Hurler and Scheie syndromes. American journal of human genetics, 53(5), 973–986.

Scott, H. S., Litjens, T., Nelson, P. V., Brooks, D. A., Hopwood, J. J., & Morris, C. P. (1992). alpha-L-iduronidase mutations (Q70X and P533R) associate with a severe Hurler phenotype. Human mutation, 1(4), 333–339. <https://doi.org/10.1002/humu.1380010412>

Sun, A., Hopwood, J. J., Thompson, J., & Cederbaum, S. D. (2011). Combined Hurler and Sanfilippo syndrome in a sibling pair. Molecular genetics and metabolism, 103(2), 135–137. <https://doi.org/10.1016/j.ymgme.2011.02.011>

Sun, L., Li, C., Song, X., Zheng, N., Zhang, H., & Dong, G. (2011). Three novel α-L-iduronidase mutations in 10 unrelated Chinese mucopolysaccharidosis type I families. Genetics and molecular biology, 34(2), 195–200. <https://doi.org/10.1590/s1415-47572011005000006>

Taghikhani, M., Khatami, S., Abdi, M., Hakhamaneshi, M. S., Alaei, M. R., Zamanfar, D., & Vakili, R. (2019). Mutation analysis and clinical characterization of Iranian patients with mucopolysaccharidosis type I. Journal of clinical laboratory analysis, 33(8), e22963. <https://doi.org/10.1002/jcla.22963>

Teng, Y. N., Wang, T. R., Hwu, W. L., Lin, S. P., & Lee-Chen, G. J. (2000). Identification and characterization of -3c-g acceptor splice site mutation in human alpha-L-iduronidase associated with mucopolysaccharidosis type IH/S. Clinical genetics, 57(2), 131–136. <https://doi.org/10.1034/j.1399-0004.2000.570207.x>

Tieu, P. T., Bach, G., Matynia, A., Hwang, M., & Neufeld, E. F. (1995). Four novel mutations underlying mild or intermediate forms of alpha-L-iduronidase deficiency (MPS IS and MPS IH/S). Human mutation, 6(1), 55–59. <https://doi.org/10.1002/humu.1380060111>

Uttarilli, A., Ranganath, P., Matta, D., Md Nurul Jain, J., Prasad, K., Babu, A. S., Girisha, K. M., Verma, I. C., Phadke, S. R., Mandal, K., Puri, R. D., Aggarwal, S., Danda, S., Sankar, V. H., Kapoor, S., Bhat, M., Gowrishankar, K., Hasan, A. Q., Nair, M., Nampoothiri, S., … Dalal, A. (2016). Identification and characterization of 20 novel pathogenic variants in 60 unrelated Indian patients with mucopolysaccharidoses type I and type II. Clinical genetics, 90(6), 496–508. <https://doi.org/10.1111/cge.12795>

Vazna, A., Beesley, C., Berna, L., Stolnaja, L., Myskova, H., Bouckova, M., Vlaskova, H., Poupetova, H., Zeman, J., Magner, M., Hlavata, A., Winchester, B., Hrebicek, M., & Dvorakova, L. (2009). Mucopolysaccharidosis type I in 21 Czech and Slovak patients: mutation analysis suggests a functional importance of C-terminus of the IDUA protein. American journal of medical genetics. Part A, 149A(5), 965–974. <https://doi.org/10.1002/ajmg.a.32812>

Venturi, N., Rovelli, A., Parini, R., Menni, F., Brambillasca, F., Bertagnolio, F., Uziel, G., Gatti, R., Filocamo, M., Donati, M. A., Biondi, A., & Goldwurm, S. (2002). Molecular analysis of 30 mucopolysaccharidosis type I patients: evaluation of the mutational spectrum in Italian population and identification of 13 novel mutations. Human mutation, 20(3), 231. <https://doi.org/10.1002/humu.9051>

Vijay, S., & Wraith, J. E. (2005). Clinical presentation and follow-up of patients with the attenuated phenotype of mucopolysaccharidosis type I. Acta paediatrica (Oslo, Norway : 1992), 94(7), 872–877. <https://doi.org/10.1111/j.1651-2227.2005.tb02004.x>

Voskoboeva, E. Y., Krasnopolskaya, X. D., Mirenburg, T. V., Weber, B., & Hopwood, J. J. (1998). Molecular genetics of mucopolysaccharidosis type I: mutation analysis among the patients of the former Soviet Union. Molecular genetics and metabolism, 65(2), 174–180. <https://doi.org/10.1006/mgme.1998.2745>

Wang, X., Zhang, W., Shi, H., Qiu, Z., Meng, Y., Yao, F., & Wei, M. (2012). Mucopolysaccharidosis I mutations in Chinese patients: identification of 27 novel mutations and 6 cases involving prenatal diagnosis. Clinical genetics, 81(5), 443–452. <https://doi.org/10.1111/j.1399-0004.2011.01680.x>

WANG, X. N., SHI, H. P., ZHANG, W. M., QIU, Z. Q., MENG, Y., YAO, F. X., & WEI, M. (2011). Zhonghua yi xue yi chuan xue za zhi = Zhonghua yixue yichuanxue zazhi = Chinese journal of medical genetics, 28(2), 147–151. <https://doi.org/10.3760/cma.j.issn.1003-9406.2011.02.006>

Wasserstein, M. P., Caggana, M., Bailey, S. M., Desnick, R. J., Edelmann, L., Estrella, L., Holzman, I., Kelly, N. R., Kornreich, R., Kupchik, S. G., Martin, M., Nafday, S. M., Wasserman, R., Yang, A., Yu, C., & Orsini, J. J. (2019). The New York pilot newborn screening program for lysosomal storage diseases: Report of the First 65,000 Infants. Genetics in medicine : official journal of the American College of Medical Genetics, 21(3), 631–640. <https://doi.org/10.1038/s41436-018-0129-y>

Yassaee, V. R., Hashemi-Gorji, F., Miryounesi, M., Rezayi, A., Ravesh, Z., Yassaee, F., & Salehpour, S. (2017). Clinical, biochemical and molecular features of Iranian families with mucopolysaccharidosis: A case series. Clinica chimica acta; international journal of clinical chemistry, 474, 88–95. <https://doi.org/10.1016/j.cca.2017.08.017>

Yogalingam, G., Guo, X. H., Muller, V. J., Brooks, D. A., Clements, P. R., Kakkis, E. D., & Hopwood, J. J. (2004). Identification and molecular characterization of alpha-L-iduronidase mutations present in mucopolysaccharidosis type I patients undergoing enzyme replacement therapy. Human mutation, 24(3), 199–207. <https://doi.org/10.1002/humu.20081>

Zahoor, M. Y., Cheema, H. A., Ijaz, S., Anjum, M. N., Ramzan, K., & Bhinder, M. A. (2019). Mapping of IDUA gene variants in Pakistani patients with mucopolysaccharidosis type 1. Journal of pediatric endocrinology & metabolism : JPEM, 32(11), 1221–1227. https://doi.org/10.1515/jpem-2019-0188
